# Deficit Symptomatology of Schizophrenia Is Associated with Attenuated Taste Identification: Findings from a Cross-Sectional Study

**DOI:** 10.3390/brainsci12111520

**Published:** 2022-11-09

**Authors:** Michał Wroński, Jerzy Samochowiec, Justyna Pełka-Wysiecka, Paweł Liśkiewicz, Przemysław Bieńkowski, Błażej Misiak

**Affiliations:** 1 Department of Psychiatry, Pomeranian Medical University, 71-460 Szczecin, Poland; 2 Department of Psychiatry, Warsaw Medical University, 02-091 Warsaw, Poland; 3 Department of Psychiatry, Division of Consultation Psychiatry and Neuroscience, Wroclaw Medical University, 50-367 Wroclaw, Poland

**Keywords:** schizophrenia, chronic illness, taste disturbances, mental disorders, deficit symptoms, dysgeusia, monosodium glutamate, MSG, taste disorder, taste perceptions

## Abstract

Schizophrenia is the subject of many studies. There have been reports of taste disturbances in mental disorders. We found a possible relationship between deficit symptoms of schizophrenia and the dysgeusia of monosodium glutamate (MSG). Dysgeusia is a disorder that distorts the sense of taste. People describe all foods as tasting sweet, sour, bitter, or metallic. We aimed to verify whether the level of MSG taste perception may be related to the severity of deficit symptoms. MSG detection threshold was assessed via sublingual administration of three fluid samples containing MSG or water. The MSG samples had different concentrations in each sample. The task was to indicate which of the samples contained MSG, determine the intensity of the taste, and assess the taste as pleasant, unpleasant, or neutral. The study group included 200 patients diagnosed with paranoid schizophrenia according to ICD-10. We found a significant negative correlation between mean intensity of taste and the number of deficit symptoms. The symptoms of taste disturbances reported by the patient should be monitored by clinicians and differentiated between the actual deficits in the field of taste perception and the taste hallucinations as a symptom of psychosis. It is important to continue research in this area.

## 1. Introduction

Schizophrenia is a chronic mental illness with periods of exacerbation and remission. As shown in the studies from the last half-century, it occurs globally with a similar frequency [1]. Using precise diagnostic methods, its lifetime prevalence has been estimated at 0.3–0.7% in the general population [2], affecting over 24 million people annually [3].

The disease manifests itself via in the presence of positive symptoms, for example, delusions and hallucinations, which result in incorrect interpretation of reality, negative symptoms, for example apathy, disturbance, or lack of social functioning, impaired motivation, and reduction in spontaneous speech. There may be cognitive impairment, for example, difficulty concentrating or problems with working memory, [4,5,6,7]. Global findings suggest it occurs 1.4 times more frequently in males relative to females, in whom the onset of symptoms is observed slightly later [8,9]. The peak incidence in men occurs before the age of 25 (15–24 years), while in women it is around the age of 30 (circa 25–34 years) [10,11]. A poorer response to pharmacological treatment is observed in men, which also results in an overall poorer prognosis as compared to women, who report fewer side effects of pharmacotherapy [12,13].

Male patients also exhibit greater manifestation of negative symptoms, not only concerning affective bluntness, apathy, impaired activity, or social isolation [14], but also confusion, problems with concentration, or memory [15]. In turn, greater severity of positive symptoms is found in women [16], and their better functioning is attributed to marriage and having children [13]. Of note, there is also early-onset schizophrenia, which occurs in children and adolescents before the age of 18, and very early-onset schizophrenia with the onset before the age of 13. The prevalence of the latter is very rare and amounts to 0.04% [17]. After the age of 14, especially in boys, it increases quite sharply and accounts for approximately 25% of all psychiatric hospitalizations in young people aged 10–18 years [18].

Currently, second-generation antipsychotics (SGA) are recommended for the treatment of schizophrenia [19,20], and the National Institute for Health and Care Excellence (NICE) additionally recommends art therapy as the method of choice and cognitive-behavioral psychotherapy (CBT) in patients with persistent negative symptoms [21]. In addition, new reports indicate that non-invasive brain stimulation (NIBS) techniques could be considered as emerging tools for enhancing clinical and/or cognitive insight in patients with schizophrenia. However, there is a need for further research in this area [22]. Available evidence shows that negative symptoms, which tend to be of chronic nature, are more difficult to treat and are present in about 20% to 40% of patients with schizophrenia [23,24].

Patients with schizophrenia frequently report numerous comorbid conditions. These include mental disorders such as depressive disorders, generalized anxiety disorder, panic disorder, or obsessive-compulsive disorder, but also somatic diseases, including cardiovascular and obstetric complications in women, hyperlipidemia, diabetes, sexual dysfunction, or osteoporosis. The presence of comorbidities may complicate the course of schizophrenia, while also affecting administered pharmacotherapy, resulting in side effects or various adverse interactions [25]. Adverse health consequences (including lung diseases) in schizophrenia patients are also reported due to their smoking status, as smoking is about three times more frequent than in the general population [26], or substance use, as substance use disorders (SUDs) are found in about 41.7% of people suffering from schizophrenia. A comorbid SUD complicates the course of the disease and its treatment, but in itself it is also difficult to treat in the population of schizophrenia patients [27].

There is a growing body of evidence that schizophrenia is a heterogenous diagnostic construct in terms of clinical manifestation and outcomes. Studies investigating this heterogeneity have demonstrated the existence of the deficit subtype of schizophrenia characterized by occurrence of primary and persistent negative symptoms. Given its genetic underpinnings, it is considered to be one of the endophenotypes of schizophrenia [28]. The prevalence of the deficit subtype has been estimated at 15% in patients with first-episode schizophrenia, and 25–30% in patients with the chronic course [29,30]. The validity of this diagnostic construct has been confirmed by functional neuroimaging studies showing increased neuronal loss, impaired glucose metabolism, and reduced blood flow to the brain of patients with deficit schizophrenia.

On the other hand, postmortem studies have demonstrated an increased density of interstitial white matter cells in their temporal and frontal cortex as compared to healthy subjects [31,32]. In the study using diffusion tensor imaging (DTI), Podwalski et al. [33] identified changes in white matter integrity (uneven distribution) in the corpus callosum of patients with the deficit syndrome. Compared to patients without the deficit syndrome and healthy controls, reduced fractional anisotropy values were found in the posterior region of their corpus callosum. Other studies have demonstrated that the prefrontal cortex and mesolimbic structures involved in the processing of odors are also associated with the pathogenesis of schizophrenia. Compared to controls, patients with schizophrenia had significantly smaller olfactory bulbs. The magnetic resonance imaging (MRI) study showed a reduced volume of the left olfactory bulb in the patients compared to their first-degree relatives, who identified the odors correctly [34]. Olfactory abnormalities described in schizophrenia have been proposed to partially reflect an existing genetic susceptibility to psychosis. In a further study [35], a reduced cortical volume was found in regions receiving signals from the olfactory bulb.

In a study using the neurovisceral integration model of fear (NVI-f), patients with more severe negative symptoms had greater difficulty in the fear conditioning in the interpersonal condition. It is believed that the fear conditioning process involves the hippocampus, amygdala, and medial prefrontal cortex, and damage to these areas of the brain may be associated with persistent negative symptoms (after the first episode of psychosis), due to reduced volume of the right hippocampus, the left amygdala, and white and gray matter in the frontal cortex. Thus, these structural abnormalities are believed to be related to the failure of fear conditioning in people with schizophrenia [36].

It is also known that the anterior cingulate cortex (ACC) is an area in the brain that regulates cognitive control. It has been found to be involved in conflict monitoring, error monitoring, attention control, and detection [37,38,39,40]. Several studies indicate that deficits in this area are associated with the occurrence of delusions in patients with schizophrenia [41,42,43,44,45].

As suggested by other reports, the ACC is an important node of the salience network (SN), responsible for identification of stimuli from the environment, as well as default-mode networks and control cognitive processes in the central executive control system [46,47,48]. Based on these reports, Zhu J. et al. (2016) found that cerebral blood flow (CBF) in the ACC gradually decreased in patients with schizophrenia (with and without delusions). Notwithstanding, similar findings were reported in healthy controls. Therefore, researchers suggest that after reaching a certain level, hypoperfusion of the ACC could lead to delusions [49,50]. In addition, they observed reduced CBF in brain regions (ACC and bilateral insular cortices) of the SN in schizophrenia patients with delusions. The conclusion was that the process of identifying stimuli as relevant may be associated with hypoperfusion of the SN, which may then lead to delusions.

Recent reports also emphasize the potential role of mitochondrial dysfunction in a number of neurodegenerative and mental diseases such as Parkinson’s diseases, Alzheimer’s disease, Huntington’s disease, amyotrophic lateral sclerosis or Friedreich’s ataxia [51], or psychiatric disorders such as schizophrenia, autism spectrum disorders, major depressive disorder (MDD), bipolar disorder, and attention-deficit hyperactive disorder [52]. Changes at the mitochondrial level are also mentioned by Tanaka et al. [53]. According to available reports, they are associated with oxidative stress and the activation of immune responses, leading to chronic inflammation, and further impairment of neuronal connectivity in the brain (frontal cortex, striatum, cerebellum) [54,55,56]. Findings also suggest abnormal glutamate-GABA concentration in the ventromedial prefrontal cortex in people diagnosed with schizophrenia. Clinical symptoms may thus be correlated with the concentration of metabolites in the anterior cingulate cortex [57]. The ventromedial prefrontal cortex (vmPFC) is involved in functions that are disturbed in schizophrenia, especially emotional tasks, which is related to vmPFC’s role in the processing of emotions [58]. This applies to social, cognitive, and affective functioning. Through interactions with ventral striatum and amygdala, vmPFC is important for decision making; through its interactions with bed nucleus of stria terminalis, amygdala, hippocampus, periaqueductal gray, and dorsal anterior cingulate cortex it contributes to generating and regulating negative emotions, while through its interactions with posterior cingulate cortex, amygdala, dorsomedial prefrontal cortex, and precuneus it is involved in social function and processing of relevant information [59]. It was also postulated that vmPFC is related to responses to tastes and flavors as a function of rated pleasantness [60,61,62,63,64]. Based on these reports, another study put forward that vmPFC may be associated with the pleasure of taste, especially the sweet taste. According to its authors, processing in this area underlies individual differences in sweet taste preference. Of note, recorded responses did not depend on the genotype, current satiety or hunger level, alcohol use, or preferred eating style. This may suggest that this region is sensitive to the perception of pleasant tastes, such as sweet taste [65].

Of note, neurodegenerative diseases (NDDs), including schizophrenia, are characterized by impairments in social interaction and cognitive function. These alterations in social functioning and cognitive performance are attributable to altered activity within subcortical and cortical brain structures [66], which store sensory, motor, and affective information, and are fundamental for decision and self-awareness processes [67]. This is a crucial aspect in the symptomatology of neurodegenerative disorders. Changing functional patterns in NDDs include impairments in emotional learning and memory, altered capacity to adapt behavior to the environment, poor planning, impaired working memory, depression, apathy, or disinhibition, which correlate with a typical cognitive pattern due to frontal lobe dysfunction [68]. Neurodegenerative processes may be linked with axonal and synaptic damage, or dendrite injury, which can lead to increased “residual” symptoms and may be associated with excessive glutamatergic transmission. In consequence, NMDA-receptor dependent excitotoxicity may take place, leading to slow neurodegeneration resulting from the opening of calcium channels, activation of intracellular enzymes and the formation of free radicals that are toxic to cell membranes and intracellular structures [69,70]. As a result, there could be an overlap between primary cognitive deficits and secondary cognitive disorders associated with progressive neurodegeneration within the central nervous system (CNS). Neurodegenerative processes are traceable in MRI studies, which show enlargement of fluid spaces in the frontal, parietal and temporal areas, as well as the lateral ventricles in patients suffering from schizophrenia [71,72,73]. The pathophysiology of schizophrenia is reported to also involve other changes related to the activity of neuropeptides and neurohormones, whose detailed descriptions are to be found elsewhere [49,74]. Much like the neurodegenerative changes in the CNS that affect the deterioration of cognitive functions, they too, perhaps, could be associated with an altered taste sensation. To resolve whether they do, however, would require further research in this regard.

Researchers highlight the role of γ-aminobutyric acid (GABA), glutamate, and dopamine, implicated both in the pathogenesis of schizophrenia and the olfactory processes [75,76]. In the study by Pełka-Wysiecka et al. [77], no significant differences in odor identification (Sniffin’ Sticks) were found between the deficit and non-deficit patients or the male and female patient groups. Interestingly, significant differences were recorded in the identification of specific odor samples. Namely, fewer deficit schizophrenia patients compared to their non-deficit counterparts correctly recognized the smell of pineapple and rose. No significant differences were found in the identification of other samples. In the male group, significantly more deficit schizophrenia patients correctly recognized the smell of orange, whereas significantly fewer correctly recognized the smell of rose. No other significant differences were found. In the female group, significant differences were observed in the identification of cinnamon. In another study, Urban-Kowalczyk et al. [78] investigated odor perception and hedonics in patients suffering from schizophrenia with the use of the University of Pennsylvania Smell Identification Test (UPSIT) and measured their blood β-endorphin (BE) and calcitonin gene-related peptide (CGRP) levels. The authors reported abnormal hedonic assessment of pleasant smells in schizophrenia patients, as well as a greater capacity to identify unpleasant odors and higher BE concentrations in people with negative symptoms. In turn, Kamath et al. [79] showed that patients diagnosed with schizophrenia made more errors in identifying pleasant and neutral smells compared to healthy controls, with no differences observed in the case of unpleasant odors. Finally, Moberg et al. [80] described reduced perception of odors among siblings of schizophrenia patients relative to healthy controls. Nevertheless, existing inconsistencies within available research results in this area warrant further investigation.

While there is evidence of smell perception deficits in patients with schizophrenia, there is a paucity of studies on dysgeusia in those with its deficit form. In his work, Faurion (1991) proposed that the receptors involved in monosodium glutamate (MSG) taste detection in the mouth may be identical to the glutamatergic receptors in the brain, which may be related to the taste sensation of MSG [81]. The taste of umami is known to be evoked in receptor cells present in both humans and animals in response to the detection of glutamate [82]. In addition, it is elicited by a number of other small molecules, including amino acids (glutamate and aspartate) and nucleotides (monosodium inosinate, disodium guanosine 5′-monophosphate, guanosine 5′-monophosphate, and 5′-monophosphate). Over the past 15 years, several receptors in the taste buds have been considered to be involved in the recognition of umami taste, including the two G protein-coupled glutamatergic receptors: metabotropic glutamate receptor 4 (mGluR4) and metabotropic glutamate receptor 1 (mGluR1) and taste bud-associated heterodimers: taste receptor type 1 member 1 + taste receptor type 3 (T1R1 + T1R3). The mGluR4 and mGluR1 receptors are activated by glutamate and its analogs, but remain insensitive to nucleotides, which, in turn, constitute activators of the T1R1 + T1 receptors, similarly to amino acids. Mice lacking the GRM4 gene manifested significantly poorer perception of the umami taste. In contrast, the elimination of the taste receptor type 1 member 1 (Tas1R1) or taste receptor type 1 member 3 (Tas1R3) genes resulted in a reduced, but not completely absent, capacity to exhibit neural and behavioral responses to this taste. To our knowledge, none of the previous studies investigated the association between deficit symptoms of schizophrenia and abnormalities in the taste perception of MSG. Therefore, the aim of this study was to find correlations between the presence of the deficit symptoms and abnormalities in the taste perception of MSG in people with schizophrenia.

## 2. Materials and Methods

### 2.1. Study Sample

The sample included 200 unrelated women and men of Polish origin diagnosed with paranoid schizophrenia (*n* = 102 and *n* = 98, respectively). The inclusion criteria were age 18–60 years, and clinical diagnosis of paranoid schizophrenia according to the International Statistical Classification of Diseases and Related Health Problems (ICD-10) criteria for at least 18 months. Each patient was examined by licensed psychiatrists during hospitalization in the Department of Psychiatry of Pomeranian Medical. Diagnosis included interview, clinical observation, PANSS score, and analyses of past medical documentation.

Upon receiving full information about the aims and the study protocol, all patients provided their written consent to participate. The protocol of this study was approved by the Bioethics Committee of the Pomeranian Medical University in Szczecin. The patients received no remuneration for participating in the study.

The exclusion criteria were as follows: a diagnosis of other mental disorders (including affective disorders), dementia or significant organic brain damage, epilepsy, addiction to alcohol or other psychoactive substances, poor general health (due to, e.g., cancer, chronic diseases of the circulatory, respiratory, digestive, or excretory systems and hormonal disorders). The adopted criteria were to enable the most precise selection of patients with paranoid schizophrenia, whose diagnosis was made following a detailed psychiatric examination, based on the ICD-10 criteria [83].

### 2.2. Assessment of Taste Detection

The patient was to refrain from smoking, consuming food, and fluids for at least one hour prior to the taste assessment. The MSG detection threshold was assessed via sublingual administration of three fluid samples (a so-called triplet), containing MSG (one sample) or water (two samples). The MSG samples had different concentrations in each triplet. Prior to the assessment and after each triplet, the patient was to rinse their mouth with water (Aqua pro injection). Assessment results were marked on a separate answer sheet. The task was to: (i) indicate which one of the three samples contained MSG—the patient’s response was recorded by the doctor on the answer sheet; (ii) determine the intensity of the taste on a scale from 0 to 100 points measured in mm (1 mm = 1 point)—the patient made an assessment on their own in the presence of the doctor; and iii) assess the taste as pleasant, unpleasant, or neutral. Pleasant tastes were to be marked on a scale from 0 to 50 points (mm), unpleasant tastes on a scale from 0 to minus 50 points, while the neutral ones were to be marked with 0—the patient made an assessment on their own in the presence of the doctor. In the experiment, we used seven triplets. In triplet 1, the first sample contained MSG at a concentration of 0.001%, while the remaining two contained water. In triplet 2, the first two samples contained water, while the third one contained MSG at a concentration of 0.003%. Triplet 3 contained water in the first and third sample, and MSG at a concentration 0.01% in the second sample. Triplet 4 contained water in the first and third sample, and MSG at a concentration of 0.03% in the second sample. Triplet 5 contained water in the first two samples, while the third sample contained 0.1% MSG. Triplet 6 contained 0.3% MSG in the first sample, while the remaining two samples contained water. In the last triplet, there was water in the first and third sample, while the second sample contained MSG at a concentration of 1%. As shown, each subsequent triplet (1–7) contained increasing concentrations of MSG.

MSG concentrations and locations in each triplet are presented in Table 1.

### 2.3. Questionnaires and Psychometric Scales

The diagnosis of paranoid schizophrenia was made after a detailed psychiatric examination, based on the ICD-10 criteria [83]. The deficit type was diagnosed using the Schedule for the Deficit Syndrome (SDS) [84]. The primary negative symptoms are chronic and form part of the disease process (i.e., are illness-related). The secondary symptoms are not intrinsic to the psychopathology of schizophrenia, but may be caused by other factors, such as, e.g., side effects of pharmacotherapy, social deprivation, environmental conditions, addiction to psychoactive substances, or comorbid affective disorders. It is, therefore, important to differentiate between them [23,85,86,87,88], which can be done using the SDS scale [89]. In our study, we used a six-point scale to assess reduced emotional expression, emotional indifference, poverty of speech, loss of interests, reduced sense of purpose, motivation, or reduced social drive. Then, we rated the severity of each examined symptom on a scale 1–4, indicated whether it was primary or secondary, and determined its stability over time, i.e., whether it was temporary or permanent (“the symptom has been present over the past 12 months and has always been present during periods of clinical stability, including chronic psychotic states”). Deficit patients were considered those that manifested symptoms of at least moderate severity (“to meet the requirements for the deficit syndrome, a patient must have a score of 2 or above on two of the six items”) [84]. The SDS is currently considered the gold standard and is therefore the most frequently used diagnostic tool. It enables differentiation between the deficit and non-deficit forms of schizophrenia, assessment of the presence and severity of negative, affective, and disorganization symptoms, quality of life, and social or professional functioning [90].

Symptomatic severity was assessed with the use of the Positive and Negative Syndrome Scale (PANSS) [91,92]. This scale assesses the severity of positive (P1–P7), negative (N1–N7), and general (G1–G16) symptoms, and each of the symptoms present were scored on a scale of 1–7 points. Sociodemographic data were collected with self-designed questionnaires.

All participants received pharmacological treatment in accordance with the current standards of care in schizophrenia [19,20,93]. All administered antipsychotic medications were converted into chlorpromazine equivalence (CPZE) [94].

### 2.4. Statistical Analysis

Descriptive statistics were presented as mean and standard deviation or the number of cases and percentage. Correlations between taste identification parameters (number of correctly recognized samples, mean intensity of taste, and mean pleasure of taste) and psychopathological symptoms (scores on the PANSS subscales and number of deficit symptoms) were tested using Spearman rank correlation coefficients. In case of significant bivariate correlations, linear regression analysis was performed to control for the effects of age, sex, body mass index (BMI), cigarette smoking status, and chlorpromazine equivalent dosage. The level of significance was set at *p* < 0.05. Data analysis was carried out using the Statistical Package for Social Sciences, version 20 (SPSS Inc., Chicago, IL, USA).

A standardized research tool (PANSS) was used to assess the course of the disease. The deficit type was distinguished using the SDS. Sociodemographic and clinical data (age, gender, BMI, cigarette smoking status, and chlorpromazine equivalent dosage) were collected with the use of self-designed questionnaires.

The characteristics of the study sample are presented in the table below (Table 2). The sample included 200 unrelated women and men of Polish origin diagnosed with paranoid schizophrenia (*n* = 102 and *n* = 98, respectively). The study sample included 98 females (48%) and 102 males (52%), whose mean age was 39.9 years. 36 persons (18%) had higher education, 94 (47%) secondary education, 44 (22%) vocational education, and the remaining 26 (13%) elementary education. Mean BMI was 27.6. The place of residence of 185 participants was the city, while the remaining 15 resided in the country. 95 participants (47.5%) reported cigarette smoking. Mean disease duration was 13.6 years. As the participants received varied pharmacological treatment, all administered antipsychotic medications were converted into chlorpromazine equivalence (CPZE) with mean dosage of 820.5 mg/day. Symptom severity was assessed with the use of the PANSS, with mean positive symptom severity of 7.7, mean negative symptom severity of 12.6, and mean depressive symptom severity of 4.3. Mean SDS score was 4.7 points.

## 3. Results

Descriptive statistics of the sample and bivariate correlations between taste identification tests and psychopathological manifestation are shown in Table 3.

In the conducted study, the following results were obtained:

- SDS scale—number of deficit symptoms: r = −0.046, *p* = 0.517 (Number of correctly recognized samples); r = −0.215, *p* = 0.002 (Mean intensity of taste); r = 0.004, *p* = 0.958 (Mean pleasure of taste)
- PANSS scale—positive symptoms: r = −0.049, *p* = 0.491 (Number of correctly recognized samples); r = −0.022, *p* = 0.760 (Mean intensity of taste); r = −0.036, *p* = 0.616 (Mean pleasure of taste)
- PANSS scale—negative symptoms: r = −0.106, *p* = 0.134 (Number of correctly recognized samples); r = −0.071, *p* = 0.322 (Mean intensity of taste); r = −0.020, *p* = 0.784 (Mean pleasure of taste)
- PANSS scale—depressive symptoms: r = −0.009, *p* = 0.896 (Number of correctly recognized samples); r = −0.029, *p* = 0.683 (Mean in-tensity of taste); r = −0.112, *p* = 0.114 (Mean pleasure of taste)

**Figure 1:**
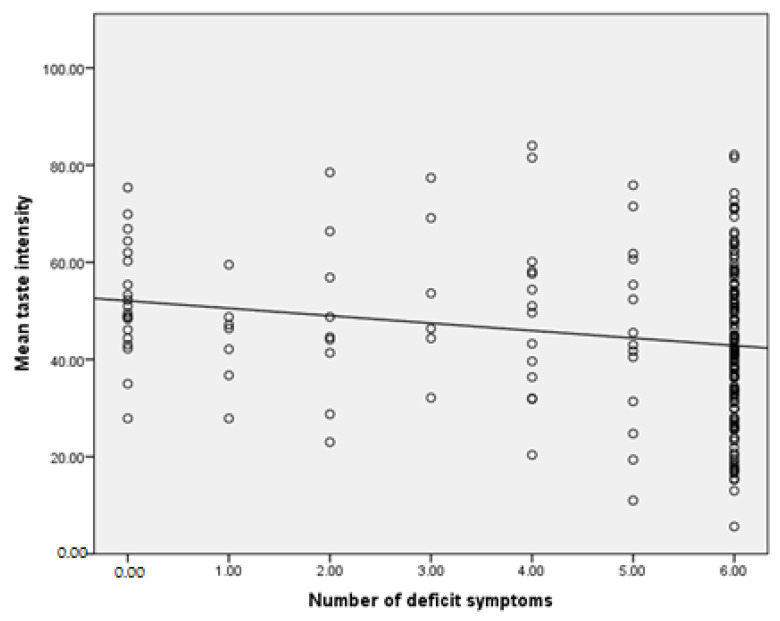
Correlation between mean taste intensity and number of deficit symptoms.

There was a significant negative correlation between mean intensity of taste and the number of deficit symptoms (Figure 1). Other measures of taste identification were not associated with the levels of psychopathological symptoms. Linear regression analysis demonstrated that this correlation remained significant (B = −1.581, SE = 0.559, *p* = 0.005) after adjustment for age (B = −0.140, SE = 0.106, *p* = 0.187), sex (B = −3.190, SE = 2.415, *p* = 0.188), BMI (B = 0.121, SE = 0.236, *p* = 0.609), cigarette smoking status (B = 0.887, SE = 2.274, *p* = 0.697), and CPZE (B = −0.001, SE = 0.001, *p* = 0.132).

Correlations between taste perception and the number of deficit symptoms are presented graphically in Figure 1 below.

## 4. Discussion

Taste disturbances that may occur in people with schizophrenia are usually overlooked in everyday clinical practice. The senses, including the sense of taste, affect perception of the surrounding world, and their disturbances in schizophrenia manifest themselves in the form of auditory, olfactory, or gustatory hallucinations. A disturbed taste recognition or assessment of its intensity does not necessarily have to be related to a mental illness and constitute a psychopathological symptom. It may be a symptom of, e.g., damage of the brain structures responsible for its perception, the taste pathways, or damage of the taste buds themselves [95].

In 1991, Faurion proposed that the receptors associated with MSG taste detection in the oral cavity may be identical to the glutamatergic receptors in the brain [81]. Therefore, we used MSG in various concentrations to determine potential taste perception disturbances in patients with deficit symptoms, considering the number of correctly recognized samples, alongside the intensity and gustatory pleasure as their measures.

There is a relative scarcity of data on MSG taste detection. What is more, available evidence applies to healthy populations. In a study conducted on a healthy Romanian population, Iordachescu et al. [96] found that 63% of their sample were unable to identify the taste of glutamate. Likewise, Singh et al. [97] reported that 3.2% of German and 4.6% of Norwegian participants did not recognize this taste at all. These studies show that MSG taste perception may be disturbed in healthy people, but no literature data have been found on the detection of MSG in people suffering from schizophrenia (or other psychoses) or affective disorders. While there is evidence that vmPFC may be associated with (especially sweet) taste perception, the study design did not evaluate the taste perception of MSG in this patient group [65].

As for clinical samples, literature data describe the perception of MSG taste in individuals with alcohol use disorders and patients with bulimia nervosa. In a study on patients with bulimia nervosa (BN), observed differences in neuronal activation of the brain after MSG administration were linked to a subjectively lower assessment of its solution in terms of both pleasure and liking of MSG compared to the control group. Functional magnetic resonance imaging (fMRI) showed greater activation of the right insula in the BN group versus the controls. Hence, BN patients exhibited not only an altered taste perception (i.e., low pleasing taste of umami), but also an altered brain activity (i.e., an altered insula function), thus explaining the disturbed eating behavior [98].

Another study considered responses to MSG in people with alcohol use disorders. The intensity and pleasure of MSG were assessed in a sample of chronic male alcoholics (*n* = 35) and compared against a control group (*n* = 25), demonstrating no significant differences [99].

Taste assessment involves a simple testing procedure that could be useful in daily practice. The fact that there are correlations between taste disturbances and symptoms of schizophrenia may provide important information for clinicians who work with psychotic patients on a daily basis. Taste disturbances reported by the patients should be monitored and properly differentiated as either actual perceptual deficits or gustatory hallucinations considered a symptom of schizophrenia. However, numerous variables (see test limitations described below) would have to be taken into account, potentially affecting test results.

In our study, we found a significant negative correlation between mean taste intensity and the number of deficit symptoms (Figure 1). Other measures of taste identification were not related to the level of psychopathological symptoms.

## 5. Conclusions

A significant negative correlation between mean intensity of taste and the number of deficit symptoms were present in this study. Other measures of taste identification (number of correctly recognized samples, mean pleasure of taste) were not associated with the levels of psychopathological symptoms.

Findings from the present study imply that deficit symptoms of schizophrenia might be associated with lower intensity of taste identification. Future studies need to further explore this observation taking into account limitations of the present study, which are described below. Longitudinal studies exploring a number of potential confounding factors and neural substrates are needed to provide additional insights into the association between altered taste identification and the deficit subtype of schizophrenia. There is no doubt that our results warrant further investigation, with an extended MSG concentration range, or on a test group with a parallel healthy control group. Extending the study sample could impact the overall results, including the number of correctly recognized samples and hedonic appraisal of the MSG taste.

A research direction to consider would also be its replication in patients suffering from other schizophrenia dimensions. Performing the same protocol in a non-paranoid subtype of schizophrenia could lead to substantially different results.

Additionally, what cannot be ignored is that the current study was cross-sectional and its results were not compared against a control group. Therefore, adding a control group could perhaps have a significant effect on the conclusions presented in this paper.

## 6. Limitations and Future Directions

The present study is characterized by certain limitations. First, we did not include healthy controls, and thus it is not possible to claim as to whether individuals with schizophrenia show deficits of taste identification. Second, there might be several latent confounding factors due to the fact that we did not record a number of potentially important clinical characteristics. These include duration and lifetime exposure to antipsychotics and other medications, as well as comorbid physical health impairments. Third, our sample may not be representative due to the fact that we did not record the initial number of patients approached for participation, as well as reasons of non-participation. Fourthly, patients recruited to the study received first and second generation antypsychotics (FGA and SGA) treatment, frequently at the same time. They were not divided into separate groups based on primary or add-on treatment (additional drugs such as antidepressants, benzodiazepines, mood stabilizers, and others). On the other hand, the assessment itself was performed at symptom remission, stable mental state, and the intake of target doses of drugs. Notwithstanding, taking medications from different groups could have had an effect on the outcomes, so we considered it amongst the limitations of this study. Fifthly, another limitation of the study was the smoking status of the participants. In order for this aspect to have as little impact on the outcomes as possible, each patient was to refrain from smoking (or consuming food or liquids) for at least one hour prior to taste assessment. Perhaps in the future, a group of non-smoking patients should be created. Sixthly, the study did not take into account whether the participants were in psychotherapy during the study. Considering this aspect, we cannot rule out that it could have an impact on the obtained results. It therefore seems that this should be considered as a limitation of our study and should be taken into account when designing similar studies in the future. Finally, a cross-sectional design does not allow for conclusions regarding causal associations.

## Figures and Tables

**Table 1:** Concentrations and locations of MSG samples in individual triplets.

Triplet No.	Sample 1	Sample 2	Sample 3
1	MSG (0.001%)	W	W
2	W	W	MSG (0.003%)
3	W	MSG (0.01%)	W
4	W	MSG (0.03%)	W
5	W	W	MSG (0, 1%)
6	MSG (0.3%)	W	W
7	W	MSG (1%)	W

MSG—monosodium glutamate; W—water.

**Table 2:** General characteristics of the sample (*n* = 200).

	Mean ± SD or *n* (%)
Age, years	39.9 ± 11.2
Sex, males	98 (48.0)
Education:	
Higher Secondary Vocational Primary	36 (18.0) 94 (47.0) 44 (22.0) 26 (13.0)
BMI, kg/m^2^	27.6 ± 4.8
Place of residence:	
Urban Rural	185 (92.5) 15 (7.5)
Cigarette smoking, yes	95 (47.5)
Illness duration, years	13.6 ± 9.2
CPZE, mg/day	820.5 ± 1179.8
PANSS—positive symptoms	7.7 ± 7.0
PANSS—negative symptoms	12.6 ± 8.8
PANSS—depressive symptoms	4.3 ± 4.0
SDS—number of deficit symptoms	4.7 ± 2.1

**Table 3:** Results of taste identification tests with respect to psychopathological manifestation.

	Number of Correctly Recognized Samples	Mean Intensity of Taste	Mean Pleasure of Taste
SDS—number of deficit symptoms	r = −0.046, *p* = 0.517	r = −0.215, *p* = 0.002	r = 0.004, *p* = 0.958
PANSS—positive symptoms	r = −0.049, *p* = 0.491	r = −0.022, *p* = 0.760	r = −0.036, *p* = 0.616
PANSS—negative symptoms	r = −0.106, *p* = 0.134	r = −0.071, *p* = 0.322	r = −0.020, *p* = 0.784
PANSS—depressive symptoms	r = −0.009, *p* = 0.896	r = −0.029, *p* = 0.683	r = −0.112, *p* = 0.114

## Data Availability

Data and materials from this study reported here are available from the corresponding author on reasonable request.

## References

[1] Häfner H., an der Heiden W. (1997). Epidemiology of Schizophrenia. Can. J. Psychiatry..

[2] Van Os J., Kapur S. (2009). Schizophrenia. Lancet.

[3] WHO (2022). Schizophrenia. http://www.who.int/mediacentre/factsheets/fs397/en/.

[4] Salazar de Pablo G., Woods S.W., Drymonitou G., de Diego H., Fusar-Poli P. (2021). Preva lence of Individuals at Clinical High-Risk of Psychosis in the General Population and Clinical Samples: Systematic Review and Meta-Analysis. Brain. Sci..

[5] American Psychiatric Association (2013). Diagnostic and Statistical Manual of Mental Disorders.

[6] Owen M.J., Sawa A., Mortensen P.B. (2016). Schizophrenia. Lancet.

[7] National Institute of Mental Health Schizophrenia. https://www.nimh.nih.gov/health/topics/schizophrenia.

[8] McGrath J., Saha S., Welham J., El Saadi O., MacCauley C., Chant D. (2004). A systematic review of the incidence of schizophrenia: The distribution of rates and the influence of sex, urbanicity, migrant status and methodology. BMC Med..

[9] Picchioni M.M., Murray R.M. (2007). Schizophrenia. BMJ.

[10] Castle D., Wessely S., Der G., Murray R.M. (1991). The incidence of operationally defined schizophrenia in Camberwell, 1965–1984. Br. J. Psychiatry.

[11] Häfner H., Maurer K., Loffler W., Riecher-Rössler A. (1993). The influence of age and sex on the onset and early course of schizophrenia. Br. J. Psychiatry.

[12] Simpson G.M., Yadalam K.G., Levinson D.F., Stephanos M.J., Sing E.E., Cooper T. (1990). Single dose pharmoacokinetics of fluphenazine after fluphenazine decanoate administation. J. Clin. Psychopharmacol..

[13] Ochoa S., Usall J., Cobo J., Labad X., Kulkarni J. (2012). Gender differences in schizophrenia and first-episode psychosis: A comprehensive literature review. Schizophr. Res. Treat..

[14] Flor-Henry P. (1990). Influence of gender in schizophrenia as related to other psychopathological syndromes. Schizophr. Bull..

[15] Maier W., Lichtermann D., Minges J., Heun R., Hallmayer J., Benkert O. (1992). Schizoaffective dis order and affective disorders with mood-incongruent psychotic features: Keep separate or combine? Evidence from a family study. Am. J. Psychiatry.

[16] Franzek B., Beckmann H. (1992). Sex differences and distinct subgroups in schizophrenia. A study of 54 chronic hospitalized schizophrenics. Psychopathology.

[17] Driver D.I., Gogtay N., Rapoport J.L. (2013). Childhood onset schizophrenia and early onset schizophrenia spectrum disorders. Child. Adolesc. Psychiatr. Clin. N. Am..

[18] Abidi S., Mian I., Garcia-Ortega I., Lecomte T., Raedler T., Jackson K., Jackson K., Pringsheim T., Addington D. (2017). Canadian guidelines for the pharmacological treatment of schizophrenia spectrum and other psychotic disorders in children and youth. Can. J. Psychiatry.

[19] Szulc A., Samochowiec J., Gałecki P., Wojnar M., Heitzman J., Dudek D. (2019). Recommendations for the treatment of schizophrenia with negative symptoms. Standards of pharmacotherapy by the Polish Psychiatric Association (Polskie Towarzystwo Psychiatryczne), part 1. Psychiatr. Pol..

[20] Szulc A., Dudek D., Samochowiec J., Wojnar M., Heitzman J., Gałecki P. (2019). Recommendations for the treatment of schizophrenia with negative symptoms. Standards of pharmacotherapy by the Polish Psychiatric Association (Polskie Towarzystwo Psychiatryczne), part 2. Psychiatr. Pol..

[21] NICE (National Institute for Health and Care Excellence) https://www.nice.org.uk/guidance/cg178/chapter/1-Recommendations#promoting-recovery-and-possible-future-care-2.

[22] Blay M., Adam O., Bation R., Galvao F., Brunelin J., Mondino M. (2022). Improvement of Insight with Non-Invasive Brain Stimulation in Patients with Schizophrenia: A Systematic Review. J. Clin. Med..

[23] Sarkar S., Hillner K., Velligan D.I. (2015). Conceptualization and treatment of negative symptoms in schizophrenia. World J. Psychiatry.

[24] Hovington C.L., Bodnar M., Joober R., Malla A.K., Lepage M. (2012). Identifying persistent negative symptoms in first episode psychosis. BMC Psychiatry.

[25] Abdullah H.M., Shahul H.A., Hwang M.Y., Ferrando S. (2020). Comorbidity in Schizophrenia: Conceptual Issues and Clinical Management. Focus.

[26] Dickerson F. (2019). Smoking and schizophrenia: Still a burning problem. Schizophr. Bull..

[27] Hunt G.E., Large M.M., Cleary M., Lai H.M.X., Saunders J.B. (2018). Prevalence of comorbid substance use in schizophrenia spectrum disorders in community and clinical settings, 1990–2017: Systematic review and meta-analysis. Drug Alcohol Depend..

[28] Samochowiec J., Pełka-Wysiecka J. (2012). Deficit schizophrenia—how to diagnose and treat?. Przew. Lek..

[29] Kirkpatrick B., Buchanan R., Ross D., Carpenter W. (2001). A separate disease within the syndrome of schizophrenia. Arch. Gen. Psychiatry.

[30] Kirkpatrick B., Fenton W., Carpenter W., Marder S. (2006). The NIMH-MATRICS consen-sus statement on negative symptoms. Schizophr. Bull.

[31] Kirkpatrick B., Conley R., Kakoyannis A., Reep R., Roberts R. (1999). Interstitial cells of the white matter in the inferior parietal cortex in schizophrenia: An unbiased cell- counting study. Synapse.

[32] Kirkpatrick B., Messias N., Conley R., Roberts R. (2003). Interstitial cells of the white matter in the dorsolateral prefrontal cortex in deficit and nondeficit schizophrenia. J. Nerv. Ment. Dis..

[33] Podwalski P., Tyburski E., Szczygieł K., Waszczuk K., Rek-Owodziń K., Mak M., Plichta P., Bielecki M., Rudkowski K., Kucharska-Mazur J. (2021). White Matter Integrity of the Corpus Callosum and Psychopathological Dimensions in Deficit and Non-Deficit Schizophrenia Patients. J. Clin. Med..

[34] Turetsky B.I., Moberg P.J., Yousem D.M., Doty R.L., Arnold S.E., Gur R.E. (2000). Reduced olfactory bulb volume in patients with schizophrenia. Am. J. Psychiatry.

[35] Turetsky B.I., Moberg P.J., Arnold S.E., Doty R.L., Gur R.E. (2003). Low olfactory bulb volume in first-degree relatives of patients with schizophrenia. Am. J. Psychiatry.

[36] Tani H., Tada M., Maeda T., Konishi M., Umeda S., Terasawa Y., Mimura M., Takahashi T., Uchida H. (2019). Comparison of emotional processing assessed with fear conditioning by interpersonal conflicts in patients with depression and schizophrenia. Psychiatry Clin. Neurosci..

[37] Silton R.L., Heller W., Towers D.N., Engels A.S., Spielberg J.M., Edgar J.C., Sass S.M., Stewart J.L., Sutton B.P., Banich M.T. (2010). The time course of activity in dorsolateral prefrontal cortex and anterior cingulate cortex during top-down attentional control. Neuroimage.

[38] Kerns J.G., Cohen J.D., MacDonald III A.W., Cho R.C., Stenger V.A., Carter C.S. (2004). Anterior cingulate conflict monitoring and adjustments in control. Science.

[39] Menon V., Adleman N.E., White C.D., Glover G.H., Reiss A.L. (2001). Error-related brain activation during a Go/NoGo response inhibition task. Hum. Brain. Mapp..

[40] Gehring W.J., Knight R.T. (2000). Prefrontal-cingulate interactions in action monitoring. Nat. Neurosci..

[41] Morris R., Griffiths O., Le Pelley M.E., Weickert T.W. (2013). Attention to irrelevant cues is related to positive symptoms in schizophrenia. Schizophr. Bull..

[42] Ilankovic L.M., Allen P.P., Engel R., Kambeitz J., Riedel M., Müller N., Hennig-Fast K. (2011). Attentional modulation of external speech attribution in patients with hallucinations and delusions. Neuropsychologia.

[43] Mlakar J., Jensterle J., Frith C.D. (1994). Central monitoring deficiency and schizophrenic symptoms. Psychol Med..

[44] Frith C.D., Done D.J. (1989). Experiences of alien control in schizophrenia reflect a disorder in the central monitoring of action. Psychol. Med..

[45] Brebion G., David A.S., Bressan R.A., Ohlsen R.I., Pilowsky L.S. (2009). Hallucinations and two types of free-recall intrusion in schizophrenia. Psychol Med..

[46] Sridharan D., Levitin D.J., Menon V. (2008). A critical role for the right fronto-insular cortex in switching between central-executive and default-mode networks. Proc. Natl. Acad. Sci. USA.

[47] Seeley W.W., Menon V., Schatzberg A.F., Keller J., Glover G.H., Kenna H., Reiss A.L., Greicius M.D. (2007). Dissociable intrinsic connectivity networks for salience processing and executive control. J. Neurosci..

[48] Menon V., Uddin L.Q. (2010). Saliency, switching, attention and control: A network model of insula function. Brain Struct. Funct..

[49] Zhu J., Zhuo C., Liu F., Xu L., Yua C. (2016). Neural substrates underlying delusions in schizophrenia. Sci. Rep..

[50] Erkwoh R., Sabri O., Steinmeyer E.M., Bull U., Sass H. (1997). Psychopathological and SPECT findings in never-treated schizophrenia. Acta. Psychiatr. Scand..

[51] Wang Y., Xu E., Musich P.R., Lin F. (2019). Mitochondrial dysfunction in neurodegenerative diseases and the potential countermeasure. CNS Neurosci. Ther..

[52] Daniels T.E., Olsen E.M., Tyrka A.R. (2020). Stress and Psychiatric Disorders: The Role of Mitochondria. Annu. Rev. Clin. Psychol..

[53] Tanaka M., Szabó A., Spekker E., Polyák H., Tóth F., Vécsei L. (2022). Mitochondrial Impairment: A Common Motif in Neuropsychiatric Presentation? The Link to the Tryptophan–Kynurenine Metabolic System. Cells.

[54] Park C., Park S.K. (2012). Molecular links between mitochondrial dysfunctions and schizophrenia. Mol. Cells.

[55] Rajasekaran A., Venkatasubramanian G., Berk M., Debnath M. (2015). Mitochondrial dysfunction in schizophrenia: Pathways, mechanisms and implications. Neurosci. Biobehav. Rev..

[56] Holper L., Ben-Shachar D., Mann J.J. (2019). Multivariate meta-analyses of mitochondrial complex I and IV in major depressive disorder, bipolar disorder, schizophrenia, Alzheimer disease, and Parkinson disease. Neuropsychopharmacology.

[57] Chen T., Wang Y., Zhang J., Wang Z., Xu J., Li Y., Yang Z., Liu D. (2017). Abnormal Concentration of GABA and Glutamate in The Prefrontal Cortex in Schizophrenia.-An in Vivo 1H-MRS Study. Shanghai Arch. Psychiatry.

[58] Fan F.M., Tan S.P., Yang F.D., Tan Y.L., Zhao Y.L., Chen N., Li B.B., Song C.S., Wang Y.H., Jin Z. (2013). Ventral medial prefrontal functional connectivity and emotion regulation in chronic schizophrenia: A pilot study. Neurosci. Bull..

[59] Hiser J., Koenigs M. (2018). The multifaceted role of ventromedial prefrontal cortex in emotion, decision-making, social cognition, and psychopathology. Biol. Psychiatry..

[60] Small D.M., Zatorre R.J., Dagher A., Evans A.C., Jones-Gotman M. (2001). Changes in brain activity related to eating chocolate: From pleasure to aversion. Brain.

[61] De Araujo I.E., Rolls E.T., Kringelbach M.L., McGlone F., Phillips N. (2003). Taste-olfactory convergence, and the representation of the pleasantness of flavour, in the human brain. Eur. J. Neurosci..

[62] Kringelbach M.L., O’Doherty J., Rolls E.T., Andrews C. (2003). Activation of the human orbitofrontal cortex to a liquid food stimulus is correlated with its subjective pleasantness. Cereb. Cortex..

[63] McClure S.M., Li J., Tomlin D., Cypert K.S., Montague L.M., Montague P.R. (2004). Neural correlates of behavioral preference for culturally familiar drinks. Neuron.

[64] Plassmann H., O’Doherty J., Shiv B., Rangel A. (2008). Marketing actions can modulate neural representations of experienced pleasantness. Proc. Natl. Acad. Sci. USA.

[65] Rudenga K.J., Small D.M. (2013). Ventromedial Prefrontal Cortex Response to Concentrated Sucrose Reflects Liking Rather Than Sweet Quality Coding. Chem. Senses.

[66] Battaglia S., Fabius J.H., Moravkova K., Fracasso A., Borgomaneri S. (2022). The Neurobiological Correlates of Gaze Perception in Healthy Individuals and Neurologic Patients. Biomedicines.

[67] Sellitto M., Terenzi D., Starita F., di Pellegrino G., Battaglia S. (2022). The Cost of Imagined Actions in a Reward-Valuation Task. Brain Sci..

[68] Battaglia S. (2022). Neurobiological advances of learned fear in humans. Adv. Clin. Exp. Med..

[69] Stahl S. (2008). Stahl’s Essential Psychopharmacology: Neuroscientific Basis and Practical Application.

[70] Gargiulo P.A., Landa De Gargiulo A.I. (2014). Glutamate and modeling of schizophrenia symptoms: Review of our Findings: 1990−2014. Pharmacol. Rep..

[71] Milev P., Ho B.C., Arndt S., Nopoulos P., Andreasen N.C. (2003). Initial magnetic resonance imaging volumetric brain measurements and outcome in schizophrenia: A prospective longitudinal study with 5-year follow-up. Biol. Psychiatry.

[72] Andreasen N.C., Nopoulos P., Magnotta V., Pierson R., Ziebell S., Ho B.C. (2011). Progressive brain change in schizophrenia: A prospective longitudinal study of first-episode schizophrenia. Biol. Psychiatry.

[73] Andreasen N.C., Liu D., Ziebell S., Vora A., Ho B.C. (2013). Relapse duration, treatment intensity, and brain tissue loss in schizophrenia: A prospective longitudinal MRI study. Am. J. Psychiatry.

[74] Tanaka M., Vécsei L. (2022). Editorial of Special Issue ‘Dissecting Neurological and Neuropsychiatric Diseases: Neurodegeneration and Neuroprotection’. Int. J. Mol. Sci..

[75] Stockhorst U., Pietrowsky R. (2004). Olfactory perception, communication, and the nose-to-brain pathway. Physiol. Behav..

[76] Kosaka T., Kosaka K. (2011). “Interneurons” in the olfactory bulb revisited. Neurosci. Res..

[77] Pełka-Wysiecka J., Wroński M., Bieńkowski P., Murawiec S., Samochowiec A., Samochowiec J. (2016). Odors identification differences in deficit and nondeficit schizophrenia. Pharmacol. Rep..

[78] Urban-Kowalczyk M., Śmigielski J., Strzelecki D. (2017). Olfactory identification in patients with schizophrenia—The influence of β-endorphin and calcitonin gene-related peptide concentrations. Eur. Psychiatry.

[79] Kamath V., Turetsky B.I., Moberg P.J. (2011). Identification of pleasant, neutral, and un-pleasant odors in schizophrenia. Psychiatry Res..

[80] Moberg P.J., Doty R.L., Turetsky B.I., Wylonis L., Cannon T.D., Acosta T.A., Gur R.E. (1996). Olfactory functioning in siblings discordent for schizophrenia. Biol. Psychiatry.

[81] Faurion A. (1991). Are umami taste receptor sites structurally related to glutamate CNS receptor sites?. Physiol. Behav..

[82] Chandrashekar J., Hoon M.A., Ryba N.J., Zuker C.S. (2006). The receptors and cells for mammalian taste. Nature.

[83] WHO (1998). International Statistical Classification of Diseases and Health Problems. Tenth Revision. Classification of Mental and Behavioral Disorders in the ICD-10. Research Diagnostic Criteria.

[84] Kirkpatrick B., Buchanan R.W., McKenney P.D., Alphs L.D., Carpenter W.T. (1989). The Schedule for the deficit syndrome: An instrument for research in schizophrenia. Psychiatry Res..

[85] Galderisi S., Mucci A., Buchanan R.W., Arango C. (2018). Negative symptoms of schizophrenia: New developments and unanswered research questions. Lancet Psychiatry.

[86] Kirschner M., Aleman A., Kaiser S. (2017). Secondary negative symptoms—A review of mechanisms, assessment and treatment. Schizophr. Res..

[87] Kirkpatrick B. (2014). Progress in the study of negative symptoms. Schizophr. Bull..

[88] Moller H.J. (2007). Clinical evaluation of negative symptoms in schizophrenia. Eur. Psychiatry.

[89] Wójciak P. (2017). Objawy negatywne schizofrenii pierwotne i wtórne, zespół deficytowy, uporczywe objawy negatywne. Neuropsychiatr. I Neuropsychol..

[90] Kalisz A., Mętel D., Daren A., Błądziński P., Kruk D., Cechnicki A. (2020). Objawy negatywne, przetrwałe objawy negatywne i zespół deficytowy a nasilenie objawów schizofrenii i poziom funkcjonowania przez 20 lat. Adv. Psychiatry Neurol..

[91] Kay S.R., Fiszbein A.O., Lewis A. (1987). The Positive and Negative Syndrome Scale (PANSS) for schizophrenia. Schizophr. Bull.

[92] Leucht S., Kissling W., Davis J.M. (2010). The PANSS should be rescaled. Schizophr. Bull.

[93] Jarema M. (2015). Standards of Pharmacological Treatment of Some Mental Disorders.

[94] Szafrański T. W Labiryncie Ekwiwalencji. Krótki, Subiektywny Przewodnik. Psychiatra. Pismo dla Praktyków. Grudzień 2014-Luty 2015 / NR 7. https://www.psychiatraonline.pl/wp-content/uploads/2017/06/PSYCHIATRA_7_eBOOK_COVERED.pdf.

[95] Bałczewska E., Nowak A. (2000). Taste Disorders—Dysgeusia.

[96] Iordachescu G., Vlasceanu G., Bleoanca I., Neagu C., Iordachescu A. (2008). Umami taste and the consumer perception. Ann. Univ. Dunarea De Jos Galati. Fascicle VI-Food Technol..

[97] Singh P.B., Schuster B., Seo H.S. (2010). Variation in umami taste perception in the German and Norwegian population. Eur. J. Clin. Nutr..

[98] Setsu R., Hirano Y., Tokunaga M., Takahashi T., Numata N., Matsumoto K., Ma-suda Y., Matsuzawa D., Iyo M., Shimizu E. (2017). Increased Subjective Distaste and Altered Insula Activity to Umami Tastant in Patients with Bulimia Nervosa. Front. Psychiatry.

[99] Wrobel E., Skrok-Wolska D., Ziolkowski M., Korkosz A., Habrat B., Woronowicz B., Kukwa A., Kostowski W., Bienkowski P., Scinska A. (2005). Taste responses to monosodium glutamate after alcohol exposure. Alcohol Alcohol..

